# HIV/AIDS: global trends, global funds and delivery bottlenecks

**DOI:** 10.1186/1744-8603-1-13

**Published:** 2005-08-01

**Authors:** Hoosen M Coovadia, Jacqui Hadingham

**Affiliations:** 1 Victor Daitz Professor of HIV/AIDS Research, Nelson R. Mandela School of Medicine, University of Kwazulu Natal, Private Bag X7 Congella, 4013, South Africa; 2 AIDS Research Co-Ordinator, Nelson R. Mandela School of Medicine, University of Kwazulu Natal, Private Bag X7 Congella, 4013, South Africa

## Abstract

Globalisation affects all facets of human life, including health and well being. The HIV/AIDS epidemic has highlighted the global nature of human health and welfare and globalisation has given rise to a trend toward finding common solutions to global health challenges. Numerous international funds have been set up in recent times to address global health challenges such as HIV.

However, despite increasingly large amounts of funding for health initiatives being made available to poorer regions of the world, HIV infection rates and prevalence continue to increase world wide. As a result, the AIDS epidemic is expanding and intensifying globally. Worst affected are undoubtedly the poorer regions of the world as combinations of poverty, disease, famine, political and economic instability and weak health infrastructure exacerbate the severe and far-reaching impacts of the epidemic.

One of the major reasons for the apparent ineffectiveness of global interventions is historical weaknesses in the health systems of underdeveloped countries, which contribute to bottlenecks in the distribution and utilisation of funds. Strengthening these health systems, although a vital component in addressing the global epidemic, must however be accompanied by mitigation of other determinants as well. These are intrinsically complex and include social and environmental factors, sexual behaviour, issues of human rights and biological factors, all of which contribute to HIV transmission, progression and mortality. An equally important factor is ensuring an equitable balance between prevention and treatment programmes in order to holistically address the challenges presented by the epidemic.

## Introduction

Globalisation, narrowly defined by Joseph Stiglitz as "the removal of barriers to free trade and the closer integration of national economies." [1], has a much wider sweep and also affects the political, cultural and social life of populations across the globe. The health sector is no exception. As Barnett and Whiteside [2] point out, health and wellbeing are international concerns and global goods, and inherent in the epidemic are lessons to be learned regarding collective responsibility for universal human health.

AIDS is a pandemic of unprecedented pervasiveness, spreading to the furthest corners of the world. Globalisation is both midwife to the spread of the disease, as modern travel facilitates rapid dissemination of HIV infection across national borders, and, through concerted global action, triumphant conqueror over its devastating impact and expansion. Despite poorer countries having ever greater access to money, effective and affordable interventions, and technical support, the epidemic continues unabated in many of the resource-constrained regions of the world. A major reason for this continued spread is the numerous constraints within health systems in developing countries, which impact upon government policy, strategic and health policy management and health service delivery.

In this paper, we discuss trends in the global AIDS epidemic as well as the numerous global funds that are available to meet the challenges posed by the disease. We also highlight the need for equal prominence to be given to both treatment and prevention programmes in the global fight against HIV/AIDS. Lastly, we examine how bottlenecks in health systems of developing countries reduce the effectiveness of such aid and suggest ways in which these blockages can be eradicated through systematic strengthening of health systems.

### Trends in the global epidemic

Despite increased resources being available to address the global AIDS challenge, the infection continues to spread. Table 1 shows the regional progression in HIV infection rates over the last five years.

**Table 1:** Trends in HIV Infections By Region

Region	No of people living with HIV (end of 1998) [39]	No of people living with HIV (end of 2003) [40]	% Increase 1998–2003
Sub-Saharan Africa	22,500,000	25,000,000	11%
South & South-East Asia	6,700,000	6,500,000	-3% ^1^
Eastern Europe & Central Asia	270,000	1,300,000	381%
Western Europe	500,000	580,000	16%
East Asia	560,000	900,000	61%
Oceania	12,000	32,000	167%
North Africa & Middle East	210,000	480,000	129%
North America	890,000	1,000,000	12%
Caribbean	330,000	430,000	30%
Latin America	1,400,000	1,600,000	14%
TOTAL	33,372,000	37,822,000	13%

^1 ^this apparent decrease is due to inconsistencies in data collection methods between earlier and later years, as well as revised estimates by UNAIDS.

HIV prevalence is intensifying in most regions, with sub-Saharan Africa, Eastern Europe and Central Asia being the worst hit, accounting for approximately 79% of new infections between 1998 and 2003. Although the greatest number of people living with HIV are in sub-Saharan Africa, of equal concern is the growing epidemic in Central Asia [3].

The epidemiology of the disease differs between regions. It has been suggested that, due to dissimilar patterns of sexual behaviour between Africa and Asia, the extent of the spread to the heterosexual population in Asia will be circumscribed. In most of sub-Saharan Africa, HIV spreads through an intricate web of relationships from sex workers to male clients to female spouses/partners. According to Peter Piot of UNAIDS, females in Africa generally report more sexual partners than their Asian counterparts [4]. In most of Central Asia transmission is virtually linear, from intravenous drug users to sex workers to male clients to female spouses/partners, with women tending to monogamy [4]. The next decade will attest to the accuracy or error of this prediction. Rising prevalence is, however, not confined to developing countries, as an increase in the number of HIV infections is evident in all other regions except South and South East Asia (where inconsistencies in data collection methods have tended to skew the figures).

### Several trends shape the HIV epidemiological curve

• An increasingly mobile global population exacerbates the risk of HIV transmission. The increasing volume of international travel contributes to the spread of sexually transmitted infections, including HIV [5]. Refugee populations arising from areas of conflict, estimated by the United Nations High Commission for Refugees to number 9,7 million worldwide [6], are at higher risk, as are internal migrants within countries, who oscillate between rural and urban milieux. According to the International Labour Organisation, at the beginning of the 21st century, 120 million workers worldwide were migrants [7].

• Females are more at risk of contracting HIV than males. In 1997, women accounted for 41% of people living with HIV worldwide. This figure had risen to almost 50% by 2002. This gender-bias is especially apparent in sub-Saharan Africa, where the majority of those infected are women and girls. Widespread wars and regional conflicts in Africa escalate, by orders of magnitude, the risk of rape of women and girls. The low social status of women, risky sexual practices, and endemic poverty in Africa contribute to the spread of the disease. The impact on women is less marked in Asia (where 28% of those infected are women), although women's low socio-economic status renders them more susceptible to infection. Women's increased vulnerability to HIV infection is not confined to developing countries. Between 2001 and 2003, the percentage of HIV-infected who are women increased in North America from 20% to 25%, and in Oceania from 17% to 19%, suggesting that gender inequalities underpin the transmission of HIV [8].

• The impact of HIV mortality is greatest on people in their 20's and 30's; this severely distorts the shape of the population pyramid in affected societies. Projections indicate that mortality rates will increase: The UN predicts that, in seven selected countries in sub-Saharan Africa, 14 million AIDS-related deaths will occur between 1995 and 2025 [9]. UNAIDS projections indicate that, unless the AIDS response is greatly increased, populations in 38 African countries will decrease by 14% by 2025 [8].

• In sub-Saharan Africa, it is estimated that 12 million children have lost one or both parents to AIDS, a figure which is expected to increase to 18 million by 2010. Even in countries where HIV infections have plateaued, the number of orphans continues to rise due to the time lapse between infection and death of parents [8].

• Agricultural output, the cornerstone of production in agrarian economies, is decreasing as a result of increased mortality in the workforce, resulting in what has been termed "new-variant famine". Studies predict that in the ten most severely affected African countries, the agricultural workforce will decline by 10–26% by 2020 [9]. Bertolt Brecht ascribed these disasters to human greed and folly: "Famines do not simply occur – they are organized by the grain trade." New-variant famine, however, is the consequence of the mutually reinforcing intercessions of human frailty and a social disease. The former from a paucity of timeous responses to the epidemic by the ruling classes, aggravated by communities steeped in stigma, fear and discrimination, and the latter from a mix of biology and human propensity to risky sexual behaviour. The combination of lost production and resulting malnutrition increase susceptibility to disease [10].

• The macroeconomic repercussions of the epidemic vary, depending on the industries underpinning the economy and degree of HIV prevalence. UNAIDS postulates that any deceleration in economic growth (as measured by Gross Domestic Product) will be offset by similar reductions in population numbers due to increased mortality and therefore resource consumption [8]. A faster decline in population size relative to GDP should theoretically result in an increase in per capita GDP. Econometric research, however, has shown that AIDS has either an insignificant impact on per capita GDP, or actually decreases it [11]. The qualitative effects of higher mortality are also considerable: the erosion of social and intellectual capital and decreased investment in populations of the future have far-reaching consequences for society as a whole [9].

• The major economic impact is microeconomic. Individual households are primarily responsible for coping with the repercussions of AIDS, and as such bear the brunt of the epidemic. This translates into increased healthcare expenses, funeral charges and education costs for households. In areas where stigma prevails, the psychological impacts of the disease increase the burden.

• Impact on the workplace is also considerable, translating into productivity losses and increased costs to employers due to staff illnesses and deaths, higher health insurance premiums and low morale [8]. In addition, household demand for goods and services may decline due to lower income and levels of consumption, resulting in the contraction of resource production [9].

Table 2 shows in summary the demographic impacts of the epidemic, while Table 3 shows the impacts on various other aspects of society. The ramifications of an epidemic of this nature and scale will be felt long after incidence of the disease has peaked, predicted in the case of HIV to be in 2040 [12]. By way of comparison, the consequences of the Black Death (1347 – 1351) extended far beyond the life of the epidemic itself, exerting influence for about 150 years in Europe [13]. In order to mitigate these effects, massive investments in prevention, treatment and care programmes and in broad development initiatives must be given priority.

**Table 2:** Summary of demographic impacts of AIDS

Demography [9]	Without AIDS	With AIDS	Without AIDS	With AIDS	Without AIDS	With AIDS
	
	1995 – 2000		2010 – 2015		2020 – 2025	
Life expectancy at birth (years)	63.9	62.4	68.4	64.2	70.8	65.9
Number of deaths (millions)	159	170	174	207	193	231
Crude death rate per 1,000	9.0	9.6	8.1	9.8	8.0	10.1
Infant mortality rate per 1,000	66.4	67.5	49.8	51.3	40.9	42.1
Child mortality rate per 1,000	93.9	98.8	68.9	75.8	56.1	62.3
Population size (millions)	3666	3639	4310	4204	4805	4599

^1,^UNAIDS Population Division, 2003

**Table 3:** Summary of sectoral impacts of AIDS

GDP [41, 42]	• Annual decrease of between 2 and 4% with AIDS
Households [9]	• Decreased household income • Increased expenditure on healthcare • More women and child-headed households • More vulnerable to poverty
Firms [9]	• Increased healthcare costs • Greater absenteeism • Loss of skilled labour and institutional memory • Decreased demand for goods → decreased income • Lower staff morale → lower productivity
Agriculture [9]	• Loss of agricultural workforce: • reduction in cultivated land → decreased yields • smaller harvest size and less crop variety • loss of agricultural knowledge • lower remittances sent home
Education [9]	• Loss of teachers → reduction in supply and quality of educational facilities and services • Increased medical and staff training costs • Reduction in pupil numbers due to non-enrolment /sickness/deaths • Reversal in progress made in primary education
Health [9]	• Absenteeism and deaths of health workers due to illness: • reduction in supply and quality of health services • increased training costs • erosion of knowledge base • Quality of care may suffer due to stigmatisation of HIV+ patients • Increased public health expenses → higher burden on private health care system • Increased demand for donor funding to address HIV/AIDS challenge • High demand for AIDS treatment crowds out treatment of other diseases

^2^Dixon, McDonald and Roberts (2002); Cornia and Zagonaria (2002)

### Global funds

Various global initiatives and collaborations are addressing the global HIV/AIDS challenge. For example, the United Nations Millennium Development Declaration, signed in 2000 by 189 nations, encompasses eight Millennium Development Goals (MDGs), three of which are health related: reducing child mortality, improving maternal health, and combating HIV/AIDS, malaria and other diseases, by 2015 [14]. Many international organizations have been set up to assist in funding and implementing HIV prevention and care programmes and related health initiatives worldwide. These include the President's Emergency Plan For AIDS Relief (PEPFAR); the Global Fund to fight AIDS, Tuberculosis and Malaria; RollBack Malaria, the Global Alliance for Vaccines and Immunization; the Global Health Council; Médecins sans Frontiers; the Bill and Melinda Gates Foundation; the World Bank Multi Country HIV/AIDS Programme (MAP); the Accelerating Access Initiative and the William J. Clinton Presidential Foundation. These organizations contribute increasing amounts of money to confront AIDS and other pressing global health issues. UNAIDS [8] reports that in 1996, approximately US$330 million was available for HIV/AIDS initiatives worldwide, a figure which had risen to US$4.7 billion by 2003. Although this represents a huge increase in funding, it is still less than half the amount of US$12 billion that is now required, and this exigency is expected to rise to US$20 billion by 2007.

Despite the large amount of aid being made available in addressing the AIDS epidemic, shortfalls in both money and numbers of people being reached are apparent. Of the estimated 6 million people in developing countries who are in need of ART, only 400,000 currently receive it. Of these, 208,000 are in Brazil alone [15]. Even if the World Health Organization's '3 by 5' effort, which aims to provide treatment to 3 million people by the end of 2005, is successful, it will have addressed only 50% of the demand for treatment *at the current level of need*. The MDGs are unlikely to be met at the current rates of progress, with the worst affected countries likely to make the least headway.

Another issue of concern is that the focus of many of these programmes is on treatment rather than prevention of HIV. Initiatives geared to increasing the delivery of treatment to developing countries has increased substantially since 2001, when the Declaration of Commitment on HIV/AIDS was signed by 189 member states of the United Nations [16]. For example:

• The Global Fund to fight AIDS, Tuberculosis and Malaria has approved funding for the provision of antiretroviral therapy (ART) to 700,000 people [17].

• The World Bank plans to increase financial assistance for ART programmes in eligible countries [17].

• PEPFAR's focus is largely on treatment [18] and plans to deliver ART to 2 million people in sub-Saharan Africa and the Caribbean by 2007 [17].

• The focus of the WHO's "3 by 5" programme is also exclusively on the treatment of HIV [15].

Current data suggests that approximately 33% of funding for AIDS initiativesbe allocated for treatment and care, with approximately 51% for prevention programmes [19]. Schwartländer et al [20] advocate a similar split in fund allocation between treatment and care on the one hand, and prevention initiatives on the other.

During the early stages of the epidemic, programmes designed to prevent HIV had rightly been the prime endeavour of poorer countries; indeed there was little else on offer. Even when the prospects of effective specific antiretroviral treatment improved after 1996, many scientists and health professionals remained committed to a dominant role of prevention over treatment and care. Prevention services, they believed, were not restricted to prophylaxis but included palliative care and the management of opportunistic infections. The latter were inexpensive and cost-effective; the concern was that highly active antiretroviral therapy (HAART), being more costly, would drain money from prevention programmes. But the direct and indirect financial, social, economic, political and security costs of failing to introduce effective prevention measures are undeniably very high. Based on figures from previous studies [21], Marseille et al modelled the cost-effectiveness of HAART against cotrimoxazole prophylaxis, and found that the ratio between the cost-effectiveness of HAART and prevention is US$350:US$12.50. (a ratio of 28:1). In human terms, for every life-year gained through HAART, 28 life-years could have been gained through prevention [22].

Marseille's evaluation, however, disregards the synergy between prevention and treatment interventions. Prevention, although an important component in addressing the epidemic, is inadequate in isolation. The low rates of uptake of preventive measures in many developing countries, which we discuss later, do not diminish this assertion. In addition to prevention programmes, the provision of HAART is not only financially feasible, but morally imperative. The difficulties associated with introducing ART are well known: there is no eradication of the virus, therefore treatment is lifelong; adherence lapses occur; drug formulations are not optimised; drug toxicities are frequent; drug-drug interactions complicate management and drug resistance requires special attention. In addition, there are aspects of HAART management which are still not settled – optimal start time and regimen sequence, the meaning of regime failure, and the sustainable reduction of resistance. The World Health Organization argues that the provision of ART, through its ability to prolong life and alleviate fears about HIV, can both change attitudes to the disease and, in combination with prevention, greatly reduce HIV transmission. It is suggested that resource-constrained countries such as Senegal, Thailand and Brazil, which introduced HAART early, are also the countries with the greatest success in controlling the epidemic. A 70% decline in AIDS-related deaths in affluent countries, where ART is available to the majority of the population, is cited to support this assertion [15].

It is becoming apparent that the advantages of ART might be offset by factors which may, on balance, fail to prevent or reduce transmission of the virus. These include disinhibition of risky sexual behaviour, the spread of drug-resistant strains, and an increased risk of exposure to HIV due to the improved survival rates of infected persons. In the context of the developing world, these putative negative impacts are likely exacerbated for several reasons:

• Early detection of HIV is rare. Patients tend to present in a state of advanced disease when viral load is high and the patient is very ill. This usually follows a period of relative good health during which maximal sexual activity and consequent high transmission of virus has occurred.

• Provision of ARVs may reduce condom use [17]

• ART efficacy may diminish as successive ARV regimes are used [23]

Despite these inherent hazards, given the continued escalation in HIV infections worldwide, it is reasonable and compassionate to attempt to achieve synergies between HAART and prevention services through their simultaneous implementation.

UNAIDS has identified a comprehensive list of prevention, treatment and care services which define standard services for HIV/AIDS control (Table 4). Most of these interventions are affordable by poor countries, either through their own budgets or from donor funds. A key issue is incorporation of applicable interventions into existing health services and programmes.

**Table 4:** Standard HIV/AIDS Interventions used by UNAIDS to measure resource needs and resource availability in low-and middle-income countries

**Prevention Interventions**
1. Mass media campaigns
2. Voluntary counseling and testing (VCT)
3. Condom social marketing
4. School-based AIDS education
5. Peer education for out-of-school youth
6. Outreach programmes for sex workers and their clients
7. Outreach programmes for men who have sex with men
8. Harm-reduction programmes for injecting users
9. Blood safety
10. Public sector condom promotion and distribution
11. Treatment of sexually transmitted infections
12. Workplace prevention programmes
13. Prevention of mother-to-child transmission
14. Post-exposure prophylaxis (PEP)
15. Safe injections
16. Universal precautions
17. Policy, advocacy, administration and research
**Care Services**
1. Palliative care
2. Diagnosis of HIV infection (HIV testing)
3. Treatment for opportunistic infections
4. Prophylaxis for opportunistic infections
5. Antiretroviral (ARV) therapy, including laboratory services for monitoring treatment
**Orphan Support**
1. Community support for orphan care
2. Orphanages
3. School fee support for orphans

UNAIDS, 2003

### Health systems capacity

An over-reliance on donor funds can reduce the long-term sustainability of aid programmes, and the reduced absorptive capacity of recipient countries for such assistance often results in bottlenecks, preventing aid packages from being used where they are most needed. As a result, despite higher levels of acceptance of AIDS by certain governments, a global climate of increased political stability and economic growth, and greater public access to information and advocacy, inequitable access to treatment and prevention persists. While challenges experienced by households and communities in terms of providing resources for home-based care are also significant hindrances to the effective delivery of care, shortcomings inherent in health systems constitute the major blocks in channeling ever-increasing amounts of aid to those most in need. It follows that inequities in the provision of healthcare services may escalate in the coming years unless efficiency is coupled with justice in the construction of national health systems.

Constraints relating to supply within health systems, including finance, information systems, human resources, drugs and logistics [14], as well as those on the demand-side, such as increased patient numbers, and stigma and discrimination among communities [8], hinder progress.

The example of introducing prevention of mother to child transmission (PMTCT) programmes, which are among the simplest and most cost-effective of anti-HIV programmes available, into national health systems, is illustrative of the challenges faced by developing countries. Single dose Nevirapine (a dose each to mother during delivery and to her newborn) is the most widely used regimen for PMTCT, having the advantages of simplicity, affordability, and effectiveness. Most programmes and agencies, including UNICEF, the Elizabeth Glaser Pediatric AIDS Foundation (EGPAF), and state authorities, have found that in developing countries, of the women who should be given ART, only a minority receive the drugs. Even fewer infants are given their prophylactic dose of Nevirapine. Until recently, experience suggested that, despite wide variations between countries, in general, of the HIV positive women attending antenatal clinics, probably < 20% received ARVs. Neff Walker [24] has estimated that, of the 2.1 million pregnant women who are HIV positive in any given year globally (excluding high-income countries), only 200,000 receive PMTCT interventions.

**Figure 1:**
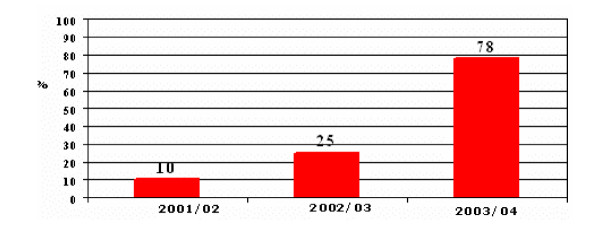
**Coverage of PMTCT Programme in Kwazulu Natal, South Africa between 2001 and 2004. **Kwazulu Natal Dept of Health (2004)

Current information from some centres, however, suggests that uptake is improving. Data from studies undertaken in Kwazulu Natal, South Africa – a region severely affected by the epidemic – show that, for 150,000 deliveries per annum, PMTCT coverage increased from 10% in 2001 to 78% in 2003/04 (Figure 1) [25]. Reasons for such improvements in a number of countries may be attributed to:

• Increased awareness of HIV due to the expansion of education, information and communication programmes, which results over time in increased acceptance of the disease and its implications. This in turn fosters greater community mobilisation in providing support groups, home-based care initiatives, orphan care and food aid.

• More rapid and reliable testing methods, including 'opt-out' options, better counseling programmes and facilities, and the inclusion of partners in both testing and counseling programmes

• Enhanced record keeping, including improved identification systems for both mothers and infants.

• Advances in drug technology and therapies with resultant wider availability of ARVs for both mothers and infants.

**Figure 2:**
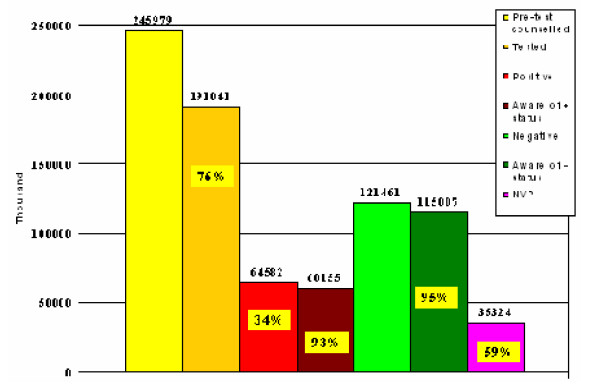
**PMTCT Uptake at maternity hospitals, clinics and community health centres in Kwazulu Natal, South Africa (June 2001 – August 2004). **Kwazulu Natal Dept of Health (2004)

Figure 2, taken from the same study, shows that despite this increase, only 59% of women attending antenatal clinics who test HIV-positive actually receive Nevirapine. Much of this attrition is due to failing health systems, although other factors, such as stigma and discrimination, also have an effect on poor uptake.

### Health system reform

The World Health Report (2004) states that "The 3 by 5 initiative...cannot be implemented in isolation from a regeneration of health systems." [26]. Several studies support this statement, reflecting the unfavourable conditions in the health care systems of developing regions [27,28].

UNAIDS [8] suggests that, in order to build capacity, an approach which incorporates training, technical assistance and access to improved guidelines and tools should be adopted by funders. In order to utilize resources effectively recipient countries need to undertake thorough planning processes whereby goals relevant to that country are set and allocation of funds is made according to need [29,30].

However, constraints may have multiple causes, both within and external to the health system itself, which may themselves be interdependent. Two approaches to overcoming constraints may be identified: dealing with constraints specific to the disease across all aspects of the health system, or addressing specific weaknesses in the health system across all diseases. It has been argued that disease-specific programmes can build skills and develop effective management structures to allow health services to cope with the demands placed on them [31]. The scale and nature of the HIV epidemic is such that it is generally the most pressing health challenge faced by developing countries. As such, an approach specific to the disease itself could be seen as the most effective way of building the capacity of health systems in countries of need, as it may be a more manageable way to address weaknesses in the health system while at the same time delivering short-term returns. This approach can, however, result in parallel systems being set up, and can cause disruptions in day to day healthcare provision. There are multiple overlaps in the health service requirements for HIV/AIDS and those for other diseases, which constitute a compelling argument to avoid as far as possible vertical schemes for HIV prevention and treatment interventions. For example, PMTCT programmes cannot be isolated from adequate antenatal clinic services, family planning, delivery facilities, and ambulatory services for chronic diseases of women and children. Indeed, the inclusion of male partners as an essential component in PMTCT-Plus indicates the broad sweep of interconnected services necessary. The frequent coinfections between HIV and tuberculosis are persuasive reasons for seeking complementarity between services for each. A system-wide response has the advantage that constraints addressed benefit a range of diseases, and draws attention to other health challenges that may be overlooked in the context of HIV/AIDS. Although the results of this approach may not be as quickly seen as in the disease-specific approach, it allows the system in its entirety to be strengthened.

It follows that the health system, rather than the specific disease, should be tackled in order to achieve the effective and holistic delivery of interventions. Such restructuring tends to be effective only in the long term, so immediate interventions may have to be introduced into the health system to deal with the pressing needs of prevention and of HIV/AIDS patients.

Robust health systems play a fundamental role in channelling globally recognised prevention and treatment best practice for the mitigation of HIV/AIDS. However, certain social and biological complexities profoundly affect the transmission, progression and mortality of the disease; these lie beyond the scope of health services. Intrinsically difficult to control, these elements constitute significant obstacles to the prevention and management of the HIV/AIDS epidemic. Biological factors, such as exposure to infected individuals (through sex, contaminated blood products, or perinatally), infectivity (determined by the viral load), and concomitant sexually transmitted infections (STIs) greatly increase susceptibility to infection. Social and environmental determinants, which include socio-economic status (for example, unemployment, poverty, degree of urbanisation and migration) may increase proclivity to risky behaviour (such as unprotected sex or drug use) and heighten the possibility of infection. Another important factor here is gender and age: women's lower status and adolescents' relative youth renders both groups more vulnerable to infection due in part to a consequent lack of power in relationships. [32-38].

It follows therefore that addressing health system constraints alone will not constitute a comprehensive solution to the management of the epidemic. Mitigation of risk factors needs to be an integral part of the response to HIV/AIDS in order for real progress to be made in the propitiation of the disease.

## Conclusion

The expansion of the AIDS epidemic across the globe has galvanized the global community into demonstrating a willingness to challenge its unabated spread. The increasing mobilisation of resources aimed at mitigating the impact of the disease in developing regions of the world in particular holds numerous potential benefits on the course of the AIDS epidemic. Whether these benefits are realized or not depends on resources dedicated to addressing the global AIDS challenge being received by those in need.

The large volumes of aid being made available to developing countries has in many instances resulted in bottlenecks in health systems in these regions, which are historically unable to cope with the demands being place upon them by the accelerating spread of HIV and concomitant influx of resources to meet this challenge. Effective delivery of aid is thus hampered. This problem can be addressed through systemic strengthening of health systems in order to build capacity and sustainability, thereby redressing the inequities in healthcare delivery due to historical differences in health systems between and within rich and poor countries. However, other risk factors such as behaviour, socio-economics and biology also contribute to the spread of the disease. It follows that addressing both health systems and these external factors is necessary in order to manage and contain HIV comprehensively.

Another important factor in the management of the epidemic is the balance between prevention and treatment programmes. The present apparent emphasis on treatment to the detriment of prevention needs to be redressed in order to meet the challenges of the disease at all levels.

Globalisation brings with it many benefits in addressing the spread of HIV throughout the world. However, these benefits can only be realized if appropriate programmes are available in areas of need. As part of the generous supply of aid aimed at addressing problems specific to HIV/AIDS, attention needs to be paid to building capacity in recipient countries so that such funds may be effectively disseminated and the epidemic effectively curbed.

## Authors' contributions

HC and JH contributed equally to the compilation of information and composition of the paper. Both authors read and approved the final manuscript
